# Supplementary material for: Empirical methods for controlling false positives and estimating confidence in ChIP-Seq peaks
Source: BMC Bioinformatics. 2008 Dec 5;9:523. doi: 10.1186/1471-2105-9-523 (PMC2628906; doi:10.1186/1471-2105-9-523)
Supplement: Additional File 1 — A variety of html documents from the USeq web site detailing the available applications, their best usage, output file type descriptions, command line menus, etc. [file 1471-2105-9-523-S1.zip › USeqUserGuides/JavaDoc.zip!/JavaDoc/igb/util/Windows2HeatMapSgr.html]

Windows2HeatMapSgr


|  |  |  |
| --- | --- | --- |
| **Overview** \| **Package** \| **Class** \| **Use** \| **Tree** \| **Deprecated** \| **Index** \| **Help** | |  |
| **PREV CLASS**   NEXT CLASS | **FRAMES**    **NO FRAMES**     **All Classes** |  |
| SUMMARY: NESTED \| FIELD \| CONSTR \| METHOD | DETAIL: FIELD \| CONSTR \| METHOD |  |


---


## igb.util Class Windows2HeatMapSgr

```
java.lang.Object
  igb.util.Windows2HeatMapSgr
```

---

``` public class Windows2HeatMapSgr extends java.lang.Object ```

Converts a text file (columns: chrom, start, stop, score) into a heat/block map specific
sgr file for import into IGB.

---

**Constructor Summary**

`Windows2HeatMapSgr(java.lang.String[] args)`


| **Method Summary** | |
| --- | --- |
| `static boolean` | `baseOverlapsWindow(int bp, igb.util.Windows2HeatMapSgr.Window win)`             Is a given base contained within the window? |
| `double` | `highestScoringWindow(int base, int currentWindowIndex)`             Looks forward at overlapping windows, returns highest score for the given bp position. |
| `static void` | `main(java.lang.String[] args)` |
| `void` | `makeBlockMapSgrFile(java.io.File file)`             Takes an array of Window |
| `void` | `parseWindowFile(java.io.File file)` |
| `static void` | `printDocs()` |
| `void` | `processArgs(java.lang.String[] args)`             This method will process each argument and assign any new varibles |

**Methods inherited from class java.lang.Object**

`equals, getClass, hashCode, notify, notifyAll, toString, wait, wait, wait`

**Constructor Detail**

### Windows2HeatMapSgr

```
public Windows2HeatMapSgr(java.lang.String[] args)
```


**Method Detail**

### makeBlockMapSgrFile

```
public void makeBlockMapSgrFile(java.io.File file)
```

:   Takes an array of Window

---


### highestScoringWindow

```
public double highestScoringWindow(int base,
                                   int currentWindowIndex)
```

:   Looks forward at overlapping windows, returns highest score for the given bp position.

---


### baseOverlapsWindow

```
public static boolean baseOverlapsWindow(int bp,
                                         igb.util.Windows2HeatMapSgr.Window win)
```

:   Is a given base contained within the window?

---


### main

```
public static void main(java.lang.String[] args)
```

---


### parseWindowFile

```
public void parseWindowFile(java.io.File file)
```

---


### processArgs

```
public void processArgs(java.lang.String[] args)
```

:   This method will process each argument and assign any new varibles

---


### printDocs

```
public static void printDocs()
```


---


|  |  |  |
| --- | --- | --- |
| **Overview** \| **Package** \| **Class** \| **Use** \| **Tree** \| **Deprecated** \| **Index** \| **Help** | |  |
| **PREV CLASS**   NEXT CLASS | **FRAMES**    **NO FRAMES**     **All Classes** |  |
| SUMMARY: NESTED \| FIELD \| CONSTR \| METHOD | DETAIL: FIELD \| CONSTR \| METHOD |  |


---
